# Automated quantification of low amplitude rhythmic contractions (LARC) during real-world urodynamics identifies a potential detrusor overactivity subgroup

**DOI:** 10.1371/journal.pone.0201594

**Published:** 2018-08-15

**Authors:** Zachary E. Cullingsworth, Brooks B. Kelly, Nicholas A. Deebel, Andrew F. Colhoun, Anna S. Nagle, Adam P. Klausner, John E. Speich

**Affiliations:** 1 Department of Mechanical and Nuclear Engineering, Virginia Commonwealth University School of Engineering, Richmond, Virginia, United States of America; 2 Department of Surgery/Division of Urology, Virginia Commonwealth University School of Medicine, Richmond, Virginia, United States of America; 3 Department of Surgery/Division of Urology Hunter Holmes McGuire Veterans Affairs Medical Center, Richmond, Virginia, United States of America; University of Alberta, CANADA

## Abstract

**Objectives:**

Detrusor overactivity (DO) is characterized by non-voiding detrusor smooth muscle contractions during the bladder filling phase and often contributes to overactive bladder. In some patients DO is observed as isolated or sporadic contractions, while in others DO is manifested as low amplitude rhythmic contractions (LARC). The aim of this study was to develop an objective method to quantify LARC frequencies and amplitudes in urodynamic studies (UDS) and identify a subgroup DO of patients with LARC.

**Methods:**

An automated Fast Fourier Transform (FFT) algorithm was developed to analyze a 205-second region of interest of retrospectively collected “real-world” UDS ending 30 seconds before voiding. The algorithm was designed to identify the three largest rhythmic amplitude peaks in vesical pressure (P_ves_) in the 1.75–6 cycle/minute frequency range. These peak P_ves_ amplitudes were analyzed to determine whether they were 1) significant (above baseline P_ves_ activity) and 2) independent (distinct from any in abdominal pressure (P_abd_) rhythm).

**Results:**

95 UDS met criteria for inclusion and were analyzed with the FFT algorithm. During a blinded visual analysis, a neurourologist/urodynamicist identified 52/95 (55%) patients as having DO. The FFT algorithm identified significant and independent (S&I) LARC in 14/52 (27%) patients with DO and 0/43 patients (0%) without DO, resulting in 100% specificity and a significant association (Fischer’s exact test, p<0.0001). The average slowest S&I LARC frequency in this DO subgroup was 3.20±0.34 cycles/min with an amplitude of 8.40±1.30 cm-H_2_O. This algorithm can analyze individual UDS in under 5 seconds, allowing real-time interpretation.

**Conclusions:**

An FFT algorithm can be applied to “real-world” UDS to automatically characterize the frequency and amplitude of underlying LARC. This algorithm identified a potential subgroup of DO patients with LARC.

## Introduction

Isolated detrusor smooth muscle strips from animals [1–3] and humans [4–6] exhibit spontaneous contractions, which often show some degree of rhythmicity [7] and have low amplitudes relative to maximal KCl-induced contractions [8]. These low amplitude rhythmic contractions (LARC) generate afferent nerve activity [1] and are elevated in the detrusor from patients with DO [6]. LARC are responsible for localized micromotion during bladder filling, and elevated micromotion has been associated with urgency [9]. In preclinical investigations, LARC have been identified in detrusor smooth muscle strips from various mammalian species [1–6] as well as in isolated whole bladders [10–13]. Although there may not be a one-to-one correlation between contractile events observed in preclinical studies and pressure fluctuations seen during UDS, the term LARC has been used for both types of events [4]. The reason is that isolated changes in vesical pressure (P_ves_) during urodynamics must be due to underlying bladder contractions. Therefore, for this study, the term LARC refers to rhythmic contractile events that are distinct from isolated or sporadic (i.e. non-rhythmic) contractions during bladder filling.

In humans, LARC has been identified in isolated human detrusor strips[4–6] and studies using catheter-mounted electrodes have shown increased LARC in women with elevated urinary urgency.[9] However, one critical research objective has been to develop methodologies to identify and characterize LARC during human urodynamic studies (UDS). To this end, recent investigations have used Fast Fourier Transforms (FFT), a mathematical technique in which complex waveforms are broken down and presented as underlying frequencies with scaled amplitudes, to characterize LARC in UDS. The first study used FFT to quantify visually identified LARC during UDS and showed a similar underlying frequency in both UDS and in isolated human detrusor strips.[4] Another study, which used a form of FFT called wavelet analysis, showed that the technique might be used to improve and automate the diagnosis of DO.[14]

Although the diagnosis of DO is relatively straightforward and based on visual identification of involuntary contractions [15], determining whether DO represents isolated, unrelated events, as opposed to coordinated rhythmic waveforms, is much more challenging, especially at amplitudes <5 cm-H_2_O [15]. We hypothesize that among the group of individuals with visually identifiable DO there is a subgroup with quantifiable LARC. The aims of the present study were 1) to develop an automated and objective FFT algorithm to identify and quantify LARC in UDS pressure data and 2) to implement the algorithm on data from patients with and without DO to determine the algorithm’s sensitivity and specificity.

For this initial investigation, we chose to use retrospectively collected UDS data (multiple indications and replete with artifacts and technical irregularities) in order to show broad applicability of our technique in “real-world” UDS. This method could ultimately be used to identify and characterize a subgroup of DO patients with LARC.

## Methods

### Patient information

**Fig 1:**
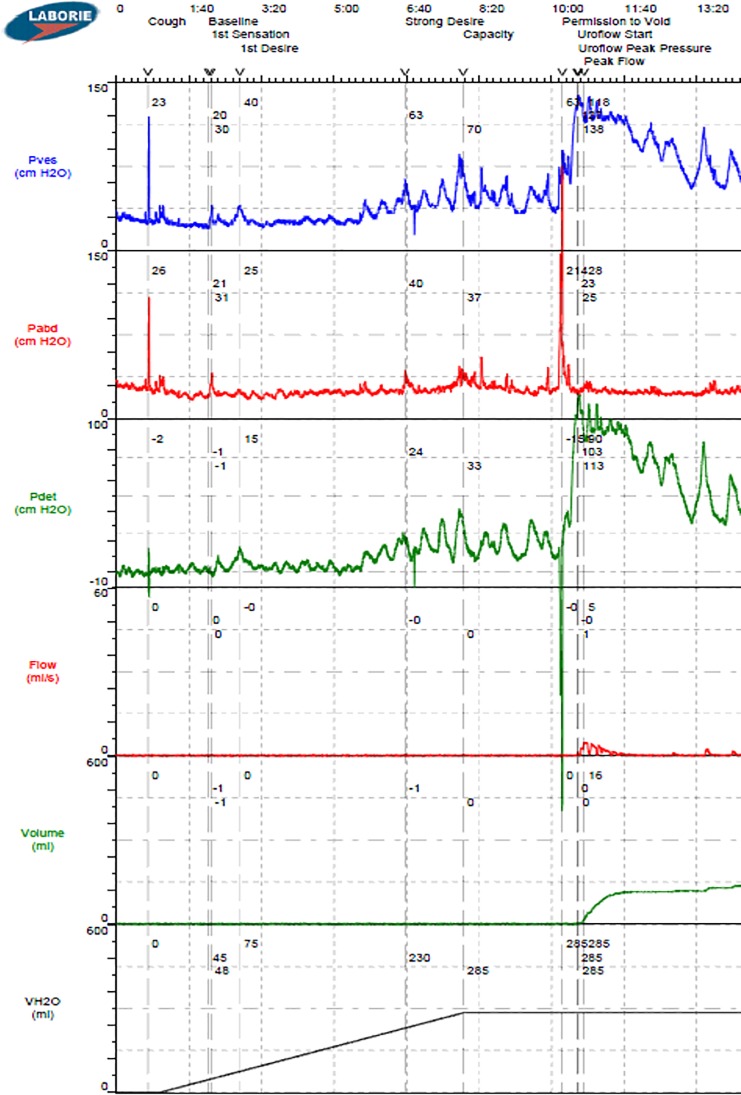
Example multichannel urodynamics output for P_ves_, P_abd_, detrusor pressure (P_det_ = P_ves_—P_abd_), infused volume (VH2O), voided volume (volume) and void flow rate (flow).

This study was approved by the Virginia Commonwealth University Institutional Review Board. Retrospective data from 239 consecutive UDS performed in the VCU Urology Urodynamics Laboratory over a three-year period were extracted directly from a Laborie Aquarius TT^TM^ multichannel UDS system (Laborie, Toronto). Data from 131 patients at least 21 years of age with complete medical records, vesical and abdominal pressure data, and at least 7 minutes of UDS filling were considered for analysis (see example in Fig 1). Studies from 36 patients with leaks or multiple voiding events were not included because the algorithm was designed to analyze a single fill-void cycle. Therefore, data from 95 studies with either a terminal voiding event or no voiding event were analyzed. The UDS pressure data were retrospectively analyzed by a neurourologist and expert urodynamicist who was blinded to other patient information. DO was diagnosed in 52 individuals based on ICS guidelines.[15] Patient characteristics as well as DO and algorithm results were then unblinded and summarized in Table 1.

**Table 1:** Patient information. P-values showing no significant association between Male vs. Female and NDO vs. IDO (Fisher’s exact test), and no significant difference in ages of S&I and DO vs. Others (t-test).

	Urologist Determination			Algorithm Determination*		
	DO	not DO	Total	S&I and DO	Others	p
**N**	52	43	95	14	38	
**Male**	26	14	40	5	35	0.8
**Female**	26	29	55	9	46	
**Age (years)**	53.4 ± 2.1	51.4 ± 2.2	52.4 ± 1.5	50.1 ± 4.1	52.9 ± 1.6	0.52
**Neurogenic**	28	17	45	8	37	0.56
**Idiopathic**	24	26	50	6	44	

*Using 30 seconds prior to void ROI

### FFT analysis of pressure data

**Fig 2:**
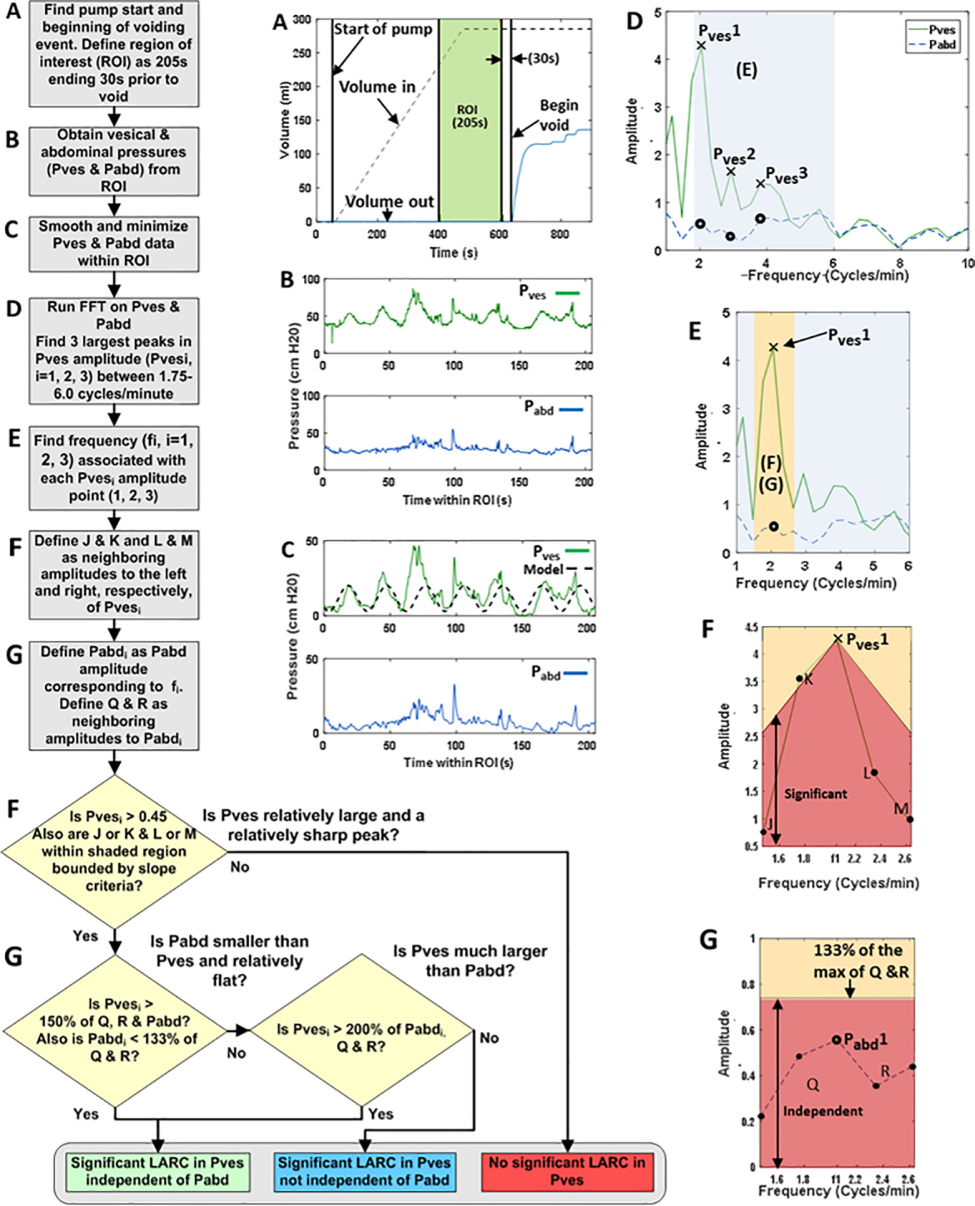
Flowchart for the FFT LARC algorithm. (A) UDS volume data showing time ROI. (B & C) raw and smoothed P_ves_ and P_abd_ data from the time ROI. (C dashed) Model of an ideal sine wave with a frequency of 2.05 cycles/min and a peak-to-peak amplitude of 17.12 cm-H2O, derived from the FFT results in panel F. The model peak-to-peak amplitude is 4 times the FFT output amplitude due to a factor of 2 from the FFT windowing and factor of 2 due to the two halves of the sine wave. (D) FFT of P_ves_ and P_abd_ from the ROI with the 1.75–6 cycle/min frequency range of interest shaded. (E) gray shaded region from panel D for P_ves_1 with range of neighboring points shaded orange. (F) Significance test: P_ves_1 is a sharp “significant” peak if J and/or K, and L and/or M are within the red region and if P_ves_1 > 0.45. (G) Independence test: a significant P_ves_1 peak is “independent” of P_abd_1 if P_abd_1 is relatively flat compared to points Q & R (within the red region below 133% of the max of Q & R) and if P_ves_1 > 150% of P_abd_1 (F & G).

**Fig 3:**
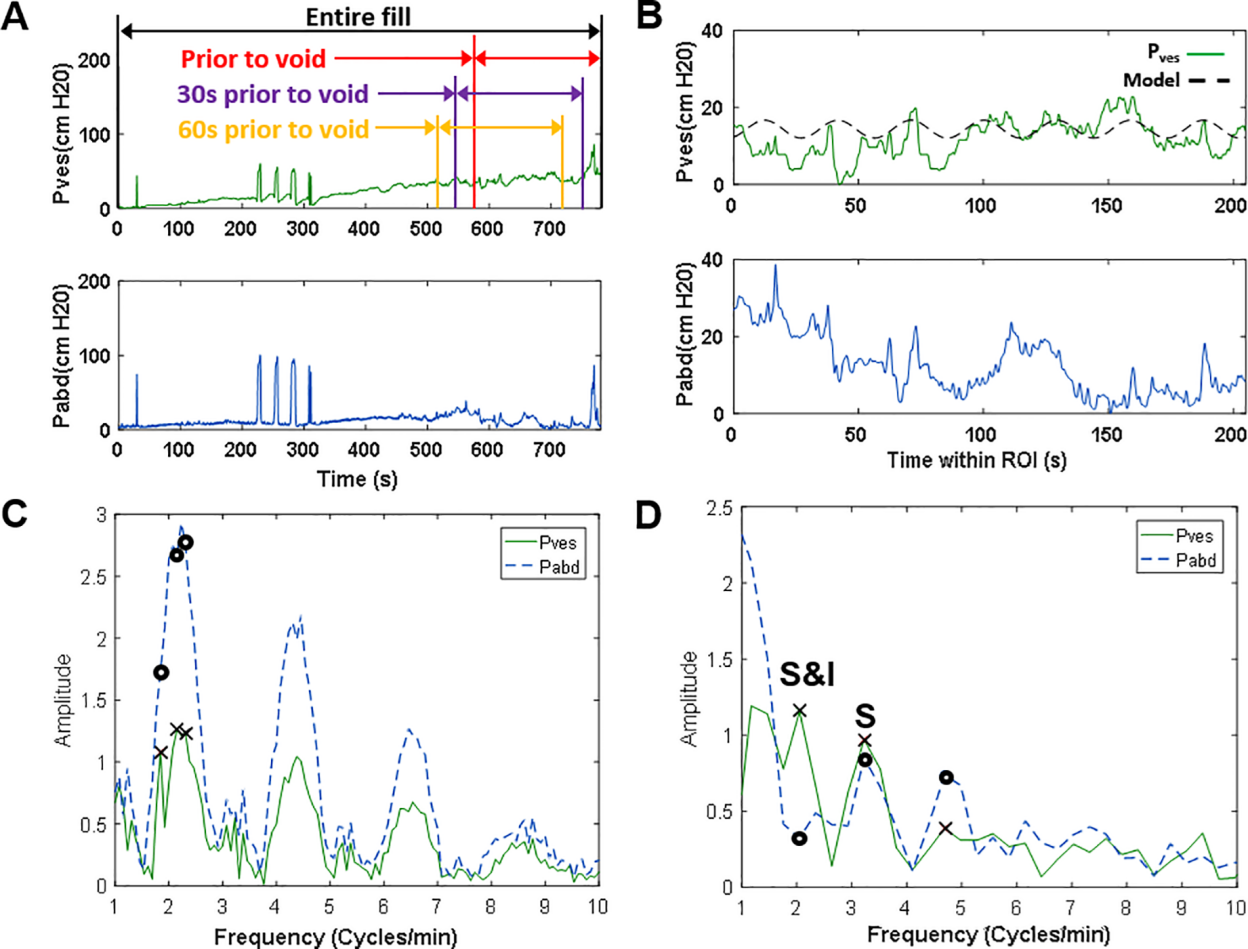
Examples of pressure tracings and corresponding FFT results. (A) Example P_ves_ and P_abd_ tracings for an entire UDS fill illustrating the 3 ROIs analyzed in this study. (B) P_ves_ and P_abd_ data for 205 seconds of filling illustrating the “30s prior to void” ROI with an overlaid sine wave model (dashed) derived from the FFT results in panel D. (C) FFT of P_ves_ and P_abd_ for the entire fill from panel A, demonstrating an increased frequency resolution (smaller spacing between the points) compared to (D), the FFT for the 205 second region in panel B with the frequency resolution used throughout this study. “S” indicates a significant P_ves_ peak that was not independent of P_abd_ and “S&I” indicates a significant P_ves_ peak independent of any corresponding P_abd_ peak.

Multichannel UDS data were loaded into an automated MATLAB (R2016A, MathWorks, Natick, MA) algorithm developed to analyze the data as described in the flow chart in Fig 2. A step-by-step example of the data analysis is provided in Fig 2 with panel labels A-G corresponding to the steps in the flow chart. The actual data from the UDS machine example (Fig 1) was utilized for the generation of Fig 2. The beginning of the voiding event, if any, was identified by analyzing the voided volume data (Fig 2A, blue line). A ROI was defined as the 205 seconds ending 30 seconds before the beginning of the voiding event (Fig 2A, green region). The 205-second duration was chosen because it represents 2048, or 2^11^, data points collected at 10 Hz, permitting consistent bin width and windowing during FFT analysis. The algorithm was implemented on three 205-second ROIs ending 1) at void, 2) 30 seconds prior to void, and 3) 60 seconds prior to void (Fig 3A, red, purple, and yellow, respectively). The reason for varying this time from the onset of voiding was because some voiding contractions (i.e. not filling phase activity) can precede the onset of voiding and because there can be some delay in the detection of voiding between the time urine leaves the urethral meatus to the time it hits the flowmeter.

P_ves_ and P_abd_ data for the ROI in its raw form (Fig 2B) were shifted by subtracting the minimum value for each signal from all data in the ROI. P_ves_ and P_abd_ data were subsequently smoothed using a 10-point moving average (Fig 2C). A Hanning window was applied to P_ves_ and P_abd_ data to eliminate discontinuities at the beginning and end of the ROI when making the signal periodic. FFT analysis was performed independently on the P_ves_ and P_abd_ data in the ROI to convert the signals to the frequency domain (Fig 2D). Normalized pressure amplitudes corresponding to frequencies within a range of 1.75–6.0 cycles/min were analyzed (Fig 2E) based on previous studies showing bladder rhythmic activity occurring between 1.8 and 5 cycles/min.[4, 5, 9].

### Significant and independent LARC

The results of the FFT analysis were used to identify LARC in P_ves_ signals with “significant” amplitude peaks at frequencies within the 1.75–6.0 cycles/min range that were “independent” of any amplitude peaks in P_abd._ Within range of 1.75–6 cycles/min, the three largest amplitude peaks in P_ves_ and corresponding frequencies were identified (Fig 2D, P_ves_1-3). For each P_ves_ peak (Fig 2E, P_ves_1), the two neighboring points on each side (Fig 2F, points J and K on left and points L and M on the right) were analyzed to test for significance (Fig 2F). In addition, in order to show independence from P_abd,_ the corresponding P_abd_ point (Fig 2G, P_abd_1) was compared with one neighboring P_abd_ point on each side (Fig 2G, points Q and R).

### LARC significance criteria

Two threshold criteria were established to determine whether each P_ves_ peak was significant. First, to eliminate P_ves_ peaks with very small amplitudes, the normalized P_ves_ amplitude was required to be greater than a threshold of 1.8 cm-H_2_O. Second, to determine whether a P_ves_ peak was of adequate prominence (sharpness), a slope criterion was established (Fig 2F, red shaded region). This slope corresponded to a steep grade (20% P_ves_ per frequency step) in order to demonstrate a sharp increase from the adjacent frequency point. To meet this criterion, point J and/or K and point L and/or M must appear in the red shaded region in Fig 2F.

### LARC independence criteria

Three thresholds were established to determine whether a significant P_ves_ peak was independent of the corresponding P_abd_ data. First, P_ves_ was required to be greater than a threshold value of 1.5 times P_abd_ and its two nearest neighboring values. This threshold was designed to identify a P_ves_ value that was relatively large compared to P_abd._ This threshold was also designed to check that the LARC in the bladder was not due to bowel contractions. Second, P_abd_ was required to be less than a threshold of 133% of its nearest neighboring values. This threshold was designed to determine that P_abd_ was less than or relatively flat compared to its neighbors and also to check that LARC in the bladder was not due to bowel contractions. Third, as a separate independence criteria, P_ves_ was considered independent of P_abd_ if P_ves_ was greater than a threshold value of two times both P_abd_ and its two nearest neighbors, regardless of any peak in P_abd_. This threshold was designed to check that significant bladder LARC was not caused by much a smaller corresponding rhythmic pressure changes noted in the bowel.

The threshold values for significance and independence of P_ves_ were determined by inspecting ~15 studies to determine values that identified signals with visible LARC. Once appropriate values were identified, the automated algorithm was implemented to analyze the entire set of 95 studies. The algorithm grouped the patients into three categories: those with 1) no significant rhythm found in P_ves_, 2) significant rhythm in P_ves_ that was not independent of P_abd_, or 3) significant rhythm in P_ves_ that was independent of P_abd_ (S&I). A patient was classified as having S&I LARC if any one or more of the three peak P_ves_ frequencies was found to be S&I.

### Statistical analysis

Fisher’s exact test was used to determine whether there was a significant association between the two groups (DO and not DO) and the two outcomes (S&I and not S&I) (Table 2). This test was also used to determine any association between male vs. females and between neurogenic DO vs. idiopathic DO (Table 1). A two-tailed, two-sample, equal variance t-test was used to determine whether there was a significant difference in the ages of the two outcome groups (Table 1). Sensitivity and specificity analysis was performed on the algorithm, noting that an ideal test would have a high specificity (only identifying LARC in patients with DO) and a specificity below 50% (highlighting the algorithm’s effectiveness to identify a DO subgroup with LARC). All continuous data are reported as means ± standard error. A p-value less than 0.05 was considered significant.

**Table 2:** Algorithm sensitivities and specificities. P-values showing significant association between S&I and DO (Fisher’s exact).

Analysis Range	Significant and Independent	Observed DO	No Observed DO	Total	p-value	Sensitivity	Specificity
**Prior to void**	S&I	16	1	17	0.0003	0.3077	0.9767
	not S&I	36	42	78			
	Total	52	43	95			
**30s prior to void**	S&I	14	0	14	**<0.0001**	**0.2692**	**1.0000**
	not S&I	38	43	81			
	Total	52	43	95			
**60s prior to void**	S&I	13	0	13	0.0002	0.2500	1.0000
	not S&I	39	43	82			
	Total	52	43	95			
**Any 3 ranges**	S&I	22	1	23	<0.0001	0.4528	0.9333
	not S&I	30	42	72			
	Total	52	43	95			

## Results

The LARC algorithm was implemented for the three ROIs (immediately prior to void, 30 seconds prior to void and 60 seconds prior to void, Fig 3A), and the results are provided in Table 2. The region 30 seconds prior to void provided 100% specificity. For this region, the algorithm identified significant and independent LARC in 14 of the 52 patients with DO and none of the 45 patients without DO, resulting in 100% specificity, 27% sensitivity and a significant association (Fischer’s exact test, p<0.0001). The average slowest S&I LARC frequency was 3.20±0.34 cycles/min with an amplitude of 8.40±1.30 cm-H_2_O in this region. Of the 14 individuals with DO identified with S&I LARC in this ROI, there were no significant differences based on sex, age or classification of DO as neurogenic or idiopathic (Table 1, p>0.05). The average infused volume for these 14 individuals was 464±58 ml, which was not different from the average infused volume of 552±31 ml for the 81 patients without S&I LARC (p>0.05). The average fill rate for all patients was 42±1 ml/min.

Additional analysis was performed to identify patients with an S&I frequency in any of the three ROIs (Table 2, “Any 3 ranges”). Out of the 52 patients with DO, 22 were identified with LARC, while 1 out of 43 patients without DO were identified with LARC, corresponding to a specificity of 93%.

## Discussion

### Clinical relevance

This study demonstrates that UDS data can be automatically analyzed for the presence of LARC using a custom-made FFT algorithm. The algorithm was purposefully designed to analyze retrospectively collected “real world” UDS data in order to demonstrate broad applicability. In addition, comparisons were made in a blinded fashion to eliminate bias. This represents a significant advance from our previous publication and first ever study showing the use of FFT in human UDS.[4] In that study, visually-identified LARC was objectively quantified, but this required direct inspection of each urodynamic tracing and the introduction of potential investigator bias.[4] The automated nature of the present algorithm eliminates this potential bias. Furthermore, the new algorithm can automatically analyze a single UDS in less than 5 sec, allowing for real-time data interpretation. Another recent study,[14] utilized wavelet analysis (as opposed to FFT) to automatically identify DO in human UDS. However, this study focused on improvement in the diagnosis of DO compared to visual inspection by the urodynamicist and did not specifically attempt to identify a DO subgroup with LARC.

The key finding of the current study is that a new DO subgroup with LARC with a frequency of 3.11±0.34 cycles/min with an amplitude of 8.24±1.24 cm-H_2_O can readily be identified with 100% specificity. The sensitivity of 27% is consistent with our expectations and demonstrates that about one quarter of DO patients are characterized as being part of a LARC subgroup. Currently, according to ICS standardization,[15] the identification of DO is subjective and limited to the presence or absence of involuntary detrusor contractions and whether or not the DO is phasic or terminal. The advantage of an automated algorithm is that it allows for an objective determination of the presence of periodic LARC, distinct from non-periodic non-voiding contractions, and for an objective quantification of LARC frequency and amplitude.

### Relation to preclinical studies

Several previous studies have used FFT and other methods to quantify LARC in preclinical animal models.[2, 3, 7, 11, 16–21] Streng *et al*. observed LARC with a slow frequency of approximately 4 cycles/minute in anesthetized rats,[18] Byrne et al, identified a slow LARC frequency of 1.8 cycles/minute in rabbit detrusor strips,[7] and Lentle *et al*. observed LARC with a frequency of 3.55 cycles/minute in isolated pig bladders.[11] In addition, Colhoun *et al*. identified an average rhythmic frequency of 1.97 cycles/minute in human detrusor strips, which was similar to the 2.34 cycles/minute frequency of UDS LARC identified in that study and the 3.11 cycles/minute identified in the present study.[4]

Spontaneous rhythmic contractions in detrusor strips typically have relatively low amplitudes of ~5–15% of a maximal KCl-induced contraction [2, 22, 23]. In the present study, the average LARC amplitude was 8.24±1.24 cm-H_2_O, and the average increase in P_det_ for a voiding contraction in the patients with S&I LARC was 82.65±16.62 cm-H_2_O (n = 12, 2 of the 14 patients with S&I LARC did not void), which corresponds to a ratio that is consistent with the preclinical findings.

### Frequency range selection

Frequencies of approximately 1.8–2.6 cycles/minute were identified in the tension tracings presented by Biers *et al*. in a study of spontaneous contractions in human detrusor strips.[5] In addition, Drake *et al*. measured micromotion between sensors placed on a balloon that was filled inside of the bladder during UDS, and frequencies of 2–5 cycles/minute were identified from their micromotion tracings.[9] Based on these studies of human bladder LARC, a frequency range of 1.75–6 cycles/minute was selected for the FFT algorithm in the current investigation. This range includes all frequencies identified in the previous studies and is slower than normal respiration.

### Selection of ROI location and duration

A ramp-up of contractile activity may occur immediately prior to voiding (Fig 3A, last 30 seconds). Therefore, three ROI locations, ending immediately prior to the start of the void, 30 seconds prior to void or 60 seconds prior to void (Fig 3A), were tested with the algorithm. In terms of duration, in order to ensure a consistent bin width/frequency resolution for all patients, a constant range of 205 seconds of data was analyzed for each patient. Analysis of an entire fill longer than 205 seconds resulted in more narrow frequency bands (compare Fig 3C to 3D) and would require different algorithm thresholds for significance and independence. Also, multiple peaks might be identified at approximately the same frequency (Fig 3C, three X symbols near 2 cycles/min), which are identified as a single frequency when using the 205 sec ROI (Fig 3D, X at 2.05 cycles/min).

UDS with at least 7 minutes of filling were selected for analysis because we originally planned to analyze and compare LARC in low and high volume regions of 3.5 minutes each. We chose 3.5 minutes because we expected to find LARC frequencies of approximately 2 cycles/min [4] and wanted to analyze a range that could include several LARC cycles. Ideal implementation of FFT analysis uses data sets with a number of points equal to a power of 2, and we chose to examine 2048 points (2^11^) taken at 10 Hz, which corresponds to 204.8 seconds, or 3.5 minutes. Furthermore, we expected that examining the latter halves of UDS fills of at least 7 minutes would ensure that the analyses were performed on data from bladders that had adequate infused volume.

### P_ves_ independence from P_abd_

**Fig 4:**
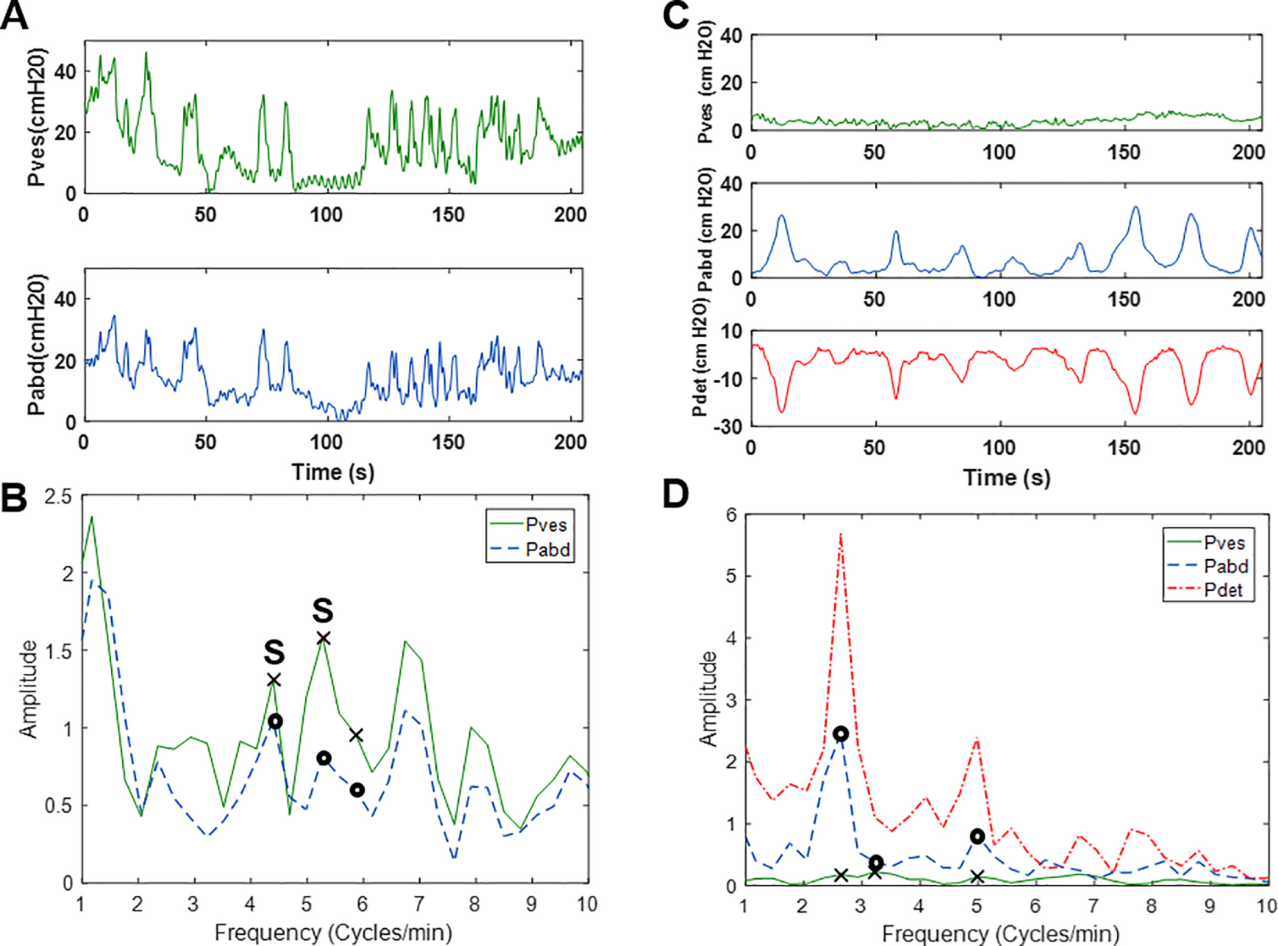
(A) Example P_ves_ and P_abd_ tracings for the final 205s of a UDS fill showing translated abdominal events and (B) the corresponding FFT output. “S” indicates a significant P_ves_ peak that was not independent of P_abd_. (C) Example P_ves_, P_abd_ and P_det_ tracings for the final 205s of a UDS fill illustrating abdominal event that was not translated to P_ves_ but appears in P_det_, and (D) the corresponding FFT output.

**Fig 5:**
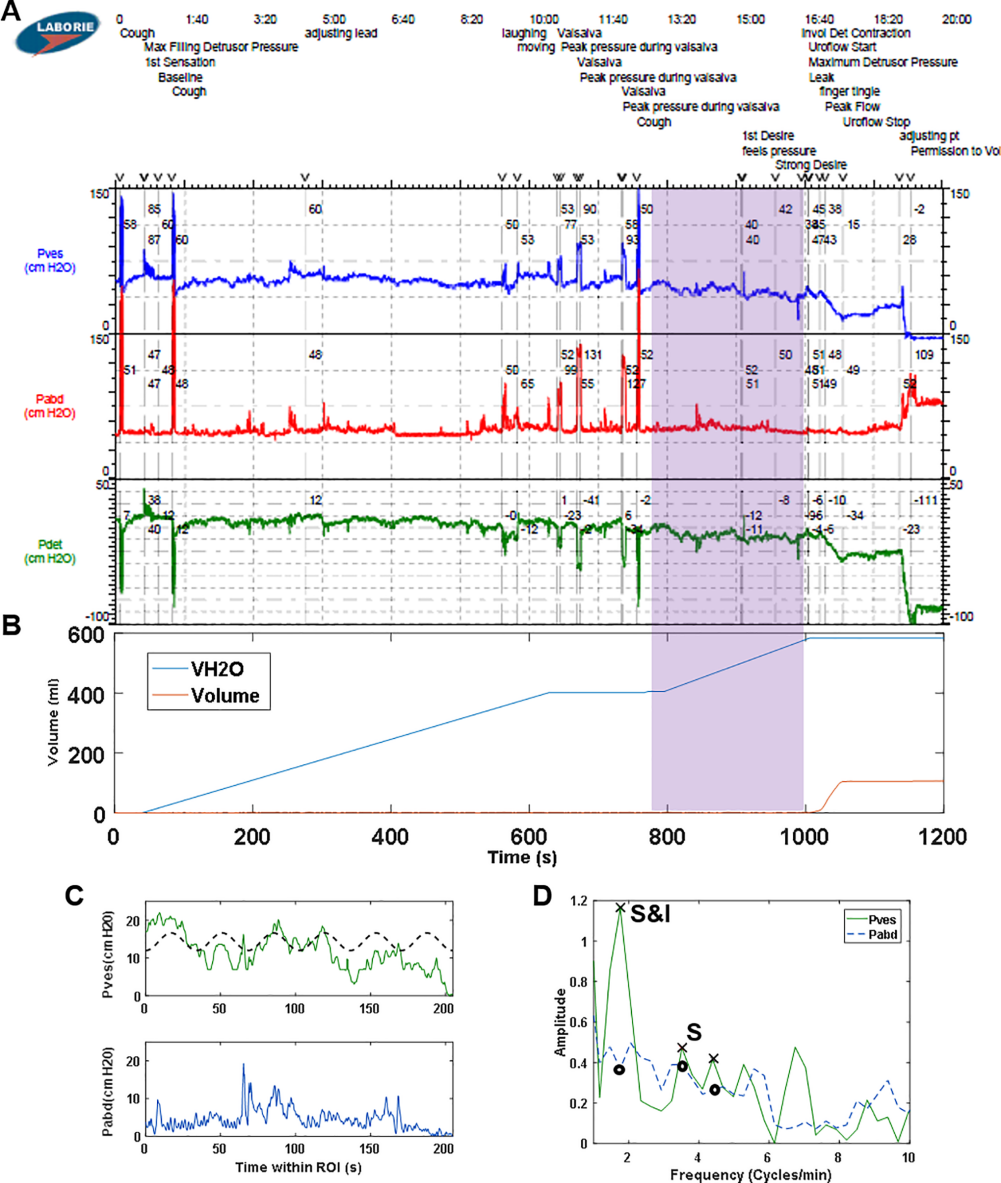
(A) Example of a UDS pressure data with P_abd_ and P_ves_ not equalized (cough at ~1.3 min) (B) corresponding infused (VH2O) and voided volumes. (C) P_ves_ and P_abd_ from the ROI (shaded region in A & B) and corresponding sine wave model (dashed) derived from FFT results in panel D. (D) FFT results of the ROI in panel C.

Each significant P_ves_ peak was compared to the corresponding P_abd_ to ensure that any bladder activity was independent of abdominal activity. For example, translated abdominal events (i.e. coughs, Valsalvas, movements) produce peaks in both the P_ves_ and P_abd_ tracings (Fig 4A), and the LARC algorithm was designed to readily identify peaks in P_ves_ that are significant but not independent of P_abd_ (Fig 4B). In addition, non-translated abdominal events (i.e. rectal contractions) produce peaks in P_abd_ and P_det_ tracings, but not P_ves_ (Fig 4C), and by analyzing P_ves_, the algorithm can determine that these events are not significant (Fig 4D). These examples provide rationale for the analysis of P_ves_ and not P_det_. Another advantage of analyzing P_ves_ and P_abd_ separately is that S&I LARC may be identified from UDS data in which P_ves_ and P_abd_ have not be completely equalized (Fig 5).

### Feasibility of implementation

The present algorithm was implemented by downloading data from the UDS machine, importing it as ASCII text into MATLAB, and implementing the custom LARC algorithm. This entire process takes only a couple of minutes, and once a data file has been loaded into MATLAB, the algorithm can analyze a single UDS and produce plots similar to those shown in Fig 4A and 4B in less than 5 seconds. In the future, this algorithm could be directly implemented using the UDS machine to provide timely analysis for clinical use. The sine wave model with the identified LARC frequency and amplitude could be displayed along with the pressure data, as in Figs 2C, 3C and 5C, to permit immediate analysis and interpretation by the clinician. Furthermore, the system could be designed to allow the clinician to select any desired ROI for LARC analysis.

### Study limitations

The present study was limited by the use of retrospective data and potential selection bias in the exclusion of studies with multiple voids/leaks (i.e. in the setting of Valsalva testing for stress urinary incontinence). However, the goal of this initial investigation was to use “real world” UDS data (performed for multiple indications and replete with artifacts and technical irregularities) to demonstrate broad applicability of the algorithm. Furthermore, the determination of “DO” was made by a neurourologist and expert urodynamicist in a blinded fashion and the algorithm was completely automated. Another issue was that the algorithm thresholds for S&I were determined iteratively using a small subset (~15 studies) of the data. In addition, because DO implies an “involuntary” contraction, the timing of a “permission to void” command is important. However, exported data from the UDS system does not include the time at which “permission to void” is granted. Incorporation of the algorithm directly into the UDS system would eliminate this limitation. A next step in the clinical implementation of this algorithm will be optimization of thresholds using prospective data obtained in different institutions and on more thoroughly characterized patient populations.

## Conclusion

This is the first study demonstrating the use of an automated FFT algorithm to identify and quantitate a DO subgroup with LARC in human UDS. The algorithm can determine if a patient with DO has periodic LARC distinct from non-periodic non-voiding contractions, identifying a DO subgroup with LARC (27% of the DO group in this study) in a matter of seconds. The algorithm was tested on retrospective UDS to be broadly applicable to real-world situations. Clinical trials using pharmacotherapies, medical devices, and behavioral treatments could be designed to analyze this unique DO subgroup with LARC to help improve treatment outcomes.

## Supporting information

S1 Data Participant ages, infused volumes, infusion rates and rhythmic contraction amplitudes and frequencies. (XLSX)
